# A unified approach to Schrödinger evolution of superoscillations and supershifts

**DOI:** 10.1007/s00028-022-00770-1

**Published:** 2022-03-14

**Authors:** Yakir Aharonov, Jussi Behrndt, Fabrizio Colombo, Peter Schlosser

**Affiliations:** 1 Schmid College of Science and Technology, Chapman University, Orange, CA 92866 USA; 2 Institut für Angewandte Mathematik, Technische Universität Graz, Steyrergasse 30, 8010 Graz, Austria; 3 Dipartimento di Matematica, Politecnico di Milano, Via E. Bonardi, 9, 20133 Milan, Italy

**Keywords:** Superoscillating function, Supershift property, Green’s function, Schrödinger equation, 81Q05, 35A08, 32A10

## Abstract

Superoscillating functions and supershifts appear naturally in weak measurements in physics. Their evolution as initial conditions in the time-dependent Schrödinger equation is an important and challenging problem in quantum mechanics and mathematical analysis. The concept that encodes the persistence of superoscillations during the evolution is the (more general) supershift property of the solution. In this paper, we give a unified approach to determine the supershift property for the solution of the time-dependent one-dimensional Schrödinger equation. The main advantage and novelty of our results is that they only require suitable estimates and regularity assumptions on the Green’s function, but not its explicit form. With this efficient general technique, we are able to treat various potentials.

## Introduction

Superoscillations are band limited functions *F* that oscillate faster than their fastest Fourier component; they appear in connection with weak measurements in quantum mechanics [2, 15, 19, 31] and as initial conditions in the time-dependent one-dimensional Schrödinger equation

1.1 $$\begin{aligned} \begin{aligned} i\frac{\partial }{\partial t}\Psi (t,x)&=\Big (-\frac{\partial ^2}{\partial x^2}+V(t,x)\Big )\Psi (t,x), \qquad t>0, x\in {\mathbb {R}},\\ \Psi (0,x)&=F(x), \end{aligned} \end{aligned}$$

or in optics, signal processing, and other fields of physics and engineering as, e.g. antenna theory [25, 43]. The theory of superoscillations and their applications has grown enormously in the last decades, and without claiming completeness, we mention the contributions [24, 26–30] and [35, 37, 38, 41, 42]. The standard example of a sequence of superoscillating functions is

1.2 $$\begin{aligned} F_n(x;k)=\sum _{l=0}^n C_l(n;k)e^{ik_l(n)x}, \end{aligned}$$

where $k>1$ and the coefficients $C_l(n;k)$, $k_l(n)$, for $n\in {\mathbb {N}}_0$, $l=0,\dots ,n$, are given by

$$\begin{aligned} C_l(n;k)={n\atopwithdelims ()l}\bigg (\frac{1+k}{2}\bigg )^{n-l}\bigg (\frac{1-k}{2}\bigg )^l\quad \text {and}\quad k_l(n)=1-\frac{2l}{n}, \end{aligned}$$

using the binomial coefficient ${n\atopwithdelims ()l}$. If we fix $x\in {\mathbb {R}}$ and let *n* go to infinity, we obtain the limit

1.3 $$\begin{aligned} \lim _{n\rightarrow \infty }F_n(x;k)=e^{ikx} \end{aligned}$$

uniformly on compact subsets of ${\mathbb {R}}$. Observe that the frequencies $k_l(n)$ in (1.2) are in modulus bounded by 1, but the frequency *k* in the limit function in (1.3) can be arbitrary large; this (somewhat unexpected) behaviour gives rise to the notion *superoscillations*. Inspired by the above example, it has been shown that also other coefficients lead to the same phenomenon, and the theory of superoscillations was extended to a larger class of functions in [7, 9, 11] and to functions of several variables in [10]. More precisely, a sequence $(F_n)_n$ of the form (1.2) is said to be *superoscillating* if $\sup _{n\in {\mathbb {N}}_0,\,l\in \{0,\dots ,n\}}|k_l(n)|<k$ for some $k>0$ and there exists a compact subset $K\subset {\mathbb {R}}$, called *superoscillation set*, such that

$$\begin{aligned} \lim \limits _{n\rightarrow \infty }\sup \limits _{x\in K}| F_n(x)-e^{i kx}|=0. \end{aligned}$$

The quantum mechanical evolution problem of superoscillations investigates the behaviour of the solution of the time-dependent Schrödinger equation (1.1) with superoscillatory initial data. It is of particular importance to understand if a frequency shift of the initial conditions at time $t=0$, as in (1.3), survives the time evolution and leads to a similar shift for the solutions at later times $t>0$. The first Schrödinger evolution problem of superoscillations that has been studied was the free particle in [8], where the solution gives a superoscillatory function in several variables. Later on, in the analysis of evolution problems with nonconstant potentials like the quantum harmonic oscillator [16, 32, 36], the electric field [12], or the uniform magnetic field [33], it turned out that the solution of the Schrödinger equation with superoscillatory initial datum does not formally belong to the class of superoscillatory functions, although a certain frequency shift still appears. These observations stimulated the notion of *supershift*, see, e.g. [4, 13, 33–35]. Very roughly speaking, it is known that the supershift property of the initial datum is stable under the time evolution of the Schrödinger equation for the above mentioned explicit cases. The analysis was based on sophisticated tools involving spaces of holomorphic functions with growth conditions, infinite-order differential operators, but required for each of the potentials the explicit form of the Green’s function. Although the Schrödinger evolution of superoscillations and supershifts was mainly studied in the one-dimensional situation (with the exceptions of the uniform magnetic field in three dimensions [33] or the constant electric field in *n* dimensions [12]), the above techniques in principle extend to the multidimensional setting, provided the Green’s function is known. For more details on superoscillations and supershifts, we also refer the reader to [3, 5, 6, 8, 13, 17, 21, 22] and the introductory papers [14, 18, 20, 23, 40].

A general approach to study the evolution of superoscillations and supershifts, which only relies on qualitative properties of the Green’s function and avoids its explicit form, does not exist so far. It is the main objective of the present paper to fill this gap; in fact, we shall provide a unified method, where we just assume regularity and growth conditions on the Green’s function. The starting point in this paper will be the (formal) representation

1.4 $$\begin{aligned} \Psi (t,x)=\int _{\mathbb {R}}G(t,x,y)F(y)\mathrm{d}y \end{aligned}$$

of the solution of the one-dimensional Schrödinger equation (1.1) via the corresponding Green’s function. The class of Green’s functions which fit into our general setting is specified in Assumption 3.1. In Sect. 2, we develop the theory of Fresnel integrals, which will then be used in Sect. 3 to give a rigorous meaning to the integral (1.4). More precisely, for a Green’s function satisfying Assumption 3.1 and an exponentially bounded holomorphic initial condition *F*, we show in Theorem 3.4 that (1.4) can be viewed as

1.5 $$\begin{aligned} \Psi (t,x)=\lim \limits _{\varepsilon \rightarrow 0^+}\int _{\mathbb {R}}e^{-\varepsilon y^2}G(t,x,y)F(y)\mathrm{d}y=e^{i\alpha }\int _{\mathbb {R}}G(t,x,ye^{i\alpha })F(ye^{i\alpha })\mathrm{d}y\nonumber \\ \end{aligned}$$

for some $\alpha \in (0,\frac{\pi }{2})$, and in certain situations under slightly stronger assumption, one even has

$$\begin{aligned} \Psi (t,x)=\lim \limits _{R_1,R_2\rightarrow \infty }\int _{-R_1}^{R_2}G(t,x,y)F(y)\mathrm{d}y; \end{aligned}$$

cf. Remark 3.5. Moreover, we prove in Theorem 3.6 that the solution $\Psi$ depends continuously on the initial datum *F*. This result will be one of the main reasons why the supershift property is stable for $t>0$. In Sect. 4, we first recall the supershift property in Definition 4.1 (in a slightly more general form than in earlier publications), and discuss its connection to the concept of superoscillations. Afterwards, we prove in Theorem 4.5 the time persistence of the supershift property for potentials *V*, where the corresponding Green’s function satisfies Assumption 3.1. In Sect. 5, we apply our main results to the one-dimensional Schrödinger equation with explicitly given potentials. Here, we consider the free particle as a warm up, and the time-dependent uniform electric field, the time-dependent harmonic oscillator, and the Pöschl–Teller potential as more sophisticated examples. In each case, we verify that the corresponding Green’s functions satisfies Assumption 3.1, and hence fits into our general setting. Therefore, for each of these potentials, we conclude the time persistence of the supershift property of the initial condition.

## Fresnel integrals

In this section, we provide some preliminary material on the so-called Fresnel integral, which will be used in our main results in the next sections. Roughly speaking, the main purpose is to make sense of integrals of the form

2.1 $$\begin{aligned} \int _{\mathbb {R}}e^{iy^2}f(y)\mathrm{d}y, \end{aligned}$$

in particular, in the case where the function *f* itself is not integrable.

### Proposition 2.1

Let $f:\Omega \rightarrow {\mathbb {C}}$ be holomorphic on an open set $\Omega \subseteq {\mathbb {C}}$, which contains the sector

2.2 $$\begin{aligned} S^+_\alpha :=\left\{ z\in {\mathbb {C}} \,|\, 0\le {\text{ Arg }}(z)\le \alpha \right\} \end{aligned}$$

for some $\alpha \in (0,\frac{\pi }{2})$. Then, the following assertions hold.

(i): If *f* satisfies the estimate
  2.3 $$\begin{aligned} |f(z)|\le Ae^{B|z|},\qquad z\in S^+_\alpha , \end{aligned}$$
  for some $A,B\ge 0$, then for every $y_0\in {\mathbb {R}}$
  2.4 $$\begin{aligned} \lim \limits _{\varepsilon \rightarrow 0^+}\int _0^\infty e^{-\varepsilon (y-y_0)^2}e^{iy^2}f(y)\mathrm{d}y=e^{i\alpha }\int _0^\infty e^{i(ye^{i\alpha })^2}f(ye^{i\alpha })\mathrm{d}y, \end{aligned}$$
  where both integrands are absolutely integrable.
(ii): If *f* satisfies the estimate
  2.5 $$\begin{aligned} |f(z)|\le Ae^{B{\text {Im}}(z)},\qquad z\in S_\alpha ^+, \end{aligned}$$
  for some $A,B\ge 0$, then
  2.6 $$\begin{aligned} \lim \limits _{R\rightarrow \infty }\int _0^Re^{iy^2}f(y)\mathrm{d}y=e^{i\alpha }\int _0^\infty e^{i(ye^{i\alpha })^2}f(ye^{i\alpha })\mathrm{d}y, \end{aligned}$$
  where the integrand on the right-hand side is absolutely integrable, and also the integrand on the left-hand side is absolutely integrable for every $R>0$.

### Proof

(i) Since the proof for arbitrary $y_0\in {\mathbb {R}}$ follows the same steps, we will restrict ourselves to $y_0=0$. We use the abbreviation $k=\tan (\alpha )>0$. For $R>0$, we consider the integration path


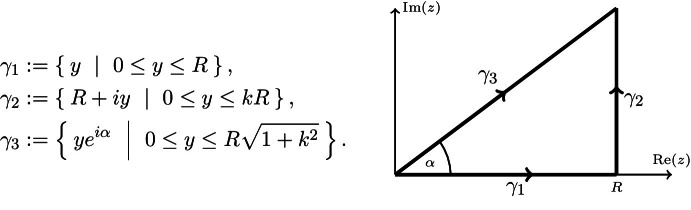


Since *f* is holomorphic, we have

2.7 $$\begin{aligned} \int _{\gamma _1}e^{(i-\varepsilon )z^2}f(z)\mathrm{d}z=-\int _{\gamma _2}e^{(i-\varepsilon )z^2}f(z)\mathrm{d}z+\int _{\gamma _3}e^{(i-\varepsilon )z^2}f(z)\mathrm{d}z. \end{aligned}$$

With the exponential bound (2.3), the integral along the curve $\gamma _2$ can be estimated as

$$\begin{aligned} \Big |\int _{\gamma _2}e^{(i-\varepsilon )z^2}f(z)\mathrm {d}z\Big |&\le A\int _0^{kR}e^{\varepsilon y^2-2Ry-\varepsilon R^2}e^{B|R+iy|}\mathrm {d}y\\&\le Ae^{-\varepsilon R^2+\sqrt{1+k^2}BR}\int _0^{kR}e^{\varepsilon y^2-2Ry}\mathrm {d}y. \end{aligned}$$

Choosing $\varepsilon \le \frac{2}{k}$, the last integral can be estimated by

$$\begin{aligned} \int _0^{kR}e^{\varepsilon y^2-2Ry}\mathrm{d}y=kR\int _0^1e^{kR^2(\varepsilon ky-2)y}\mathrm{d}y\le kR, \end{aligned}$$

and hence we conclude the convergence

$$\begin{aligned} \lim \limits _{R\rightarrow \infty }\int _{\gamma _2}e^{(i-\varepsilon )z^2}f(z)\mathrm{d}z=0. \end{aligned}$$

Consequently, in the limit $R\rightarrow \infty$ (2.7) becomes

$$\begin{aligned} \int _0^\infty e^{(i-\varepsilon )y^2}f(y)\mathrm{d}y=e^{i\alpha }\int _0^\infty e^{(i-\varepsilon )(ye^{i\alpha })^2}f(ye^{i\alpha })\mathrm{d}y, \end{aligned}$$

where the integrand on the left-hand side is absolutely integrable due to the factor $e^{-\varepsilon y^2}$, but also the right-hand side is absolutely integrable due to the estimates

$$\begin{aligned} \big |e^{(i-\varepsilon )(ye^{i\alpha })^2}f(ye^{i\alpha })\big |&\le Ae^{-(\sin (2\alpha )+\varepsilon \cos (2\alpha ))y^2+By}\\&=Ae^{-\sin (2\alpha )\big (1+\varepsilon \frac{\cos ^2(\alpha )-\sin ^2(\alpha )}{2\sin (\alpha )\cos (\alpha )}\big )y^2+By} \\&\le Ae^{-\sin (2\alpha )(1-\frac{\varepsilon k}{2})y^2+By}\\&\le Ae^{-\sin (2\alpha )\frac{y^2}{2}+By} \end{aligned}$$

for all $\varepsilon <\frac{1}{k}$. Since this upper bound does not depend on $\varepsilon$, we can apply the dominated convergence theorem and obtain

$$\begin{aligned} \lim \limits _{\varepsilon \rightarrow 0^+}\int _0^\infty e^{(i-\varepsilon )y^2}f(y)\mathrm{d}y=e^{i\alpha }\int _0^\infty e^{i(ye^{i\alpha })^2}f(ye^{i\alpha })\mathrm{d}y, \end{aligned}$$

which is (2.4).

(ii) In order to prove (2.6), we use the same integration path as in (i). We get the statement from (2.5) and from the fact that for every $R>\frac{B}{2}$ we have the estimate

$$\begin{aligned} \Big |\int _{\gamma _2}e^{iz^2}f(z)\mathrm{d}z\Big |\le \int _0^{kR}\Big |e^{i(R+iy)^2}f(R+iy)\Big |\mathrm{d}y\le A\int _0^\infty e^{-(2R-B)y}\mathrm{d}y=\frac{A}{2R-B}. \end{aligned}$$

Hence, we also in this case we conclude

$$\begin{aligned} \lim \limits _{R\rightarrow \infty }\int _{\gamma _2}e^{iz^2}f(z)\mathrm{d}z=0. \end{aligned}$$

Moroever, from (2.7) with $\varepsilon =0$, we obtain

$$\begin{aligned} \lim \limits _{R\rightarrow \infty }\int _0^Re^{iy^2}f(y)\mathrm{d}y=e^{i\alpha }\int _0^\infty e^{i(ye^{i\alpha })^2}f(ye^{i\alpha })\mathrm{d}y \end{aligned}$$

where the integrand on the right-hand side is absolutely integrable due to the estimate

$$\begin{aligned} \big |e^{i(ye^{i\alpha })^2}f(ye^{i\alpha })\big |\le Ae^{-\sin (2\alpha )y^2+B\sin (\alpha )y}. \end{aligned}$$

$$\square $$

The next corollary is a slight extension of Proposition 2.1 which includes some additional constants $a>0$ and $y_1\in {\mathbb {R}}$.

### Corollary 2.2

Let $a>0$, $y_1\in {\mathbb {R}}$ and let $f:\Omega \rightarrow {\mathbb {C}}$ be holomorphic on an open set $\Omega \subseteq {\mathbb {C}}$, which contains the sector $S_\alpha ^+$ from (2.2) for some $\alpha \in (0,\frac{\pi }{2})$. Then, the following assertions hold.

(i): If *f* satisfies the estimate
  2.8 $$\begin{aligned} |f(z)|\le Ae^{B|z|},\qquad z\in S_\alpha ^+, \end{aligned}$$
  for some $A,B\ge 0$, then for every $y_0\in {\mathbb {R}}$
  2.9 $$\begin{aligned} \lim \limits _{\varepsilon \rightarrow 0^+}\int _0^\infty e^{-\varepsilon (y-y_0)^2}e^{ia(y-y_1)^2}f(y)\mathrm {d}y=e^{i\alpha }\int _0^\infty e^{ia(ye^{i\alpha }-y_1)^2}f(ye^{i\alpha })\mathrm {d}y,\nonumber \\ \end{aligned}$$
  where both integrands are absolutely integrable.
(ii): If *f* satisfies the estimate
  2.10 $$\begin{aligned} |f(z)|\le Ae^{B{\text {Im}}(z)},\qquad z\in S_\alpha ^+, \end{aligned}$$
  for some $A,B\ge 0$, then
  2.11 $$\begin{aligned} \lim \limits _{R\rightarrow \infty }\int _0^Re^{ia(y-y_1)^2}f(y)\mathrm{d}y=e^{i\alpha }\int _0^\infty e^{ia(ye^{i\alpha }-y_1)^2}f(ye^{i\alpha })\mathrm{d}y, \end{aligned}$$
  where the integrand on the right-hand side is absolutely integrable, and also the integrand on the left-hand side is absolutely integrable for every $R>0$.

### Proof

(i): Substituting $x=y\sqrt{a}$, we can write
  $$\begin{aligned} \,\,\qquad \int _0^\infty e^{-\varepsilon (y-y_0)^2}e^{ia(y-y_1)^2}f(y)\mathrm{d}y=\frac{1}{\sqrt{a}}\int _0^\infty e^{-\frac{\varepsilon }{a}(x-\sqrt{a}y_0)^2}e^{i(x-\sqrt{a}y_1)^2}f\Big (\frac{x}{\sqrt{a}}\Big )\mathrm{d}x. \end{aligned}$$

If we define $g(z):= e^{i(-2z\sqrt{a}y_1+ay_1^2)}f(\frac{z}{\sqrt{a}})$, $z\in \Omega$, we can write this integral as

2.12 $$\begin{aligned} \int _0^\infty e^{-\varepsilon (y-y_0)^2}e^{ia(y-y_1)^2}f(y)\mathrm{d}y=\frac{1}{\sqrt{a}}\int _0^\infty e^{-\frac{\varepsilon }{a}(x-\sqrt{a}y_0)^2}e^{ix^2}g(x)\mathrm{d}x. \end{aligned}$$

Since, by (2.8), *g* satisfies the estimate

$$\begin{aligned} |g(z)|=e^{2{\text {Im}}(z)\sqrt{a}y_1}\Big |f\Big (\frac{z}{\sqrt{a}}\Big )\Big |\le Ae^{\big (2\sqrt{a}|y_1|+\frac{B}{\sqrt{a}}\big )|z|},\qquad z\in S_\alpha ^+, \end{aligned}$$

we know by Proposition 2.1 (i) that

$$\begin{aligned}&\lim \limits _{\varepsilon \rightarrow 0^+}\int _0^\infty e^{-\varepsilon (y-y_0)^2}e^{ia(y-y_1)^2}f(y)\mathrm{d}y\\&\quad =\frac{1}{\sqrt{a}}\lim \limits _{\varepsilon \rightarrow 0^+}\int _0^\infty e^{-\frac{\varepsilon }{a}(x-\sqrt{a}y_0)^2}e^{ix^2}g(x)\mathrm{d}x \\&\quad =\frac{1}{\sqrt{a}}e^{i\alpha }\int _0^\infty e^{i(xe^{i\alpha })^2}g(xe^{i\alpha })\mathrm{d}x \\&\quad =e^{i\alpha }\int _0^\infty e^{ia(ye^{i\alpha }-y_1)^2}f(ye^{i\alpha })\mathrm{d}y. \end{aligned}$$

(ii): In the same way as in (2.12) for $\varepsilon =0$, we can write
  $$\begin{aligned} \int _0^Re^{ia(y-y_1)^2}f(y)\mathrm{d}y=\frac{1}{\sqrt{a}}\int _{0}^{R\sqrt{a}}e^{ix^2}g(x)\mathrm{d}x. \end{aligned}$$

Since, by (2.10), *g* satisfies the estimate

$$\begin{aligned} |g(z)|=e^{2{\text {Im}}(z)\sqrt{a}y_1}\Big |f\Big (\frac{z}{\sqrt{a}}\Big )\Big |\le Ae^{\bigl (2\sqrt{a}|y_1|+\frac{B}{\sqrt{a}}\bigr ){\text {Im}}(z)},\qquad z\in S_\alpha ^+, \end{aligned}$$

we know by Proposition 2.1 (ii) that

$$\begin{aligned} \lim \limits _{R\rightarrow \infty }\int _0^Re^{ia(y-y_1)^2}f(y)\mathrm{d}y&=\frac{1}{\sqrt{a}}\lim \limits _{R\rightarrow \infty }\int _0^{R\sqrt{a}}e^{ix^2}g(x)\mathrm{d}x \\&=\frac{1}{\sqrt{a}}e^{i\alpha }\int _0^\infty e^{i(xe^{i\alpha })^2}g(xe^{i\alpha })\mathrm{d}x \\&=e^{i\alpha }\int _0^\infty e^{ia(ye^{i\alpha }-y_1)^2}f(ye^{i\alpha })\mathrm{d}y. \end{aligned}$$

$$\square $$

The Fresnel integral technique in Proposition 2.1 and Corollary 2.2 can also be applied on the negative semi-axis. This leads to the following corollary.

### Corollary 2.3

Let $a>0$, $y_1\in {\mathbb {R}}$ and let $f:\Omega \rightarrow {\mathbb {C}}$ be holomorphic on an open set $\Omega \subseteq {\mathbb {C}}$, which contains the double sector

2.13 $$\begin{aligned} S_\alpha :=\bigl \{ z\in {\mathbb {C}}\,|\, {\text{ Arg }}(z)\in [0,\alpha ]\cup [\pi ,\pi +\alpha ]\bigr \} \end{aligned}$$

for some $\alpha \in (0,\frac{\pi }{2})$. Then, the following assertions hold.

(i): If *f* satisfies the estimate
  2.14 $$\begin{aligned} |f(z)|\le Ae^{B|z|},\qquad z\in S_\alpha , \end{aligned}$$
  for some $A,B\ge 0$, then for every $y_0\in {\mathbb {R}}$
  2.15 $$\begin{aligned} \lim \limits _{\varepsilon \rightarrow 0^+}\int _{\mathbb {R}}e^{-\varepsilon (y-y_0)^2}e^{ia(y-y_1)^2}f(y)\mathrm{d}y=e^{i\alpha }\int _{\mathbb {R}}e^{ia(ye^{i\alpha }-y_1)^2}f(ye^{i\alpha })\mathrm{d}y, \end{aligned}$$
  where both integrands are absolutely integrable.
(ii): If *f* satisfies the estimate
  2.16 $$\begin{aligned} |f(z)|\le Ae^{B|{\text {Im}}(z)|},\qquad z\in S_\alpha , \end{aligned}$$
  for some $A,B\ge 0$, then
  2.17 $$\begin{aligned} \lim \limits _{R_1,R_2\rightarrow \infty }\int _{-R_1}^{R_2}e^{ia(y-y_1)^2}f(y)\mathrm{d}y=e^{i\alpha }\int _{\mathbb {R}}e^{ia(ye^{i\alpha }-y_1)^2}f(ye^{i\alpha })\mathrm{d}y, \end{aligned}$$
  where the integrand on the right-hand side is absolutely integrable, and also the integrand on the left-hand side is absolutely integrable for every $R_1,R_2>0$.

The initial purpose of the Fresnel integral technique was to give meaning to the integral (2.1). For holomorphic functions *f* satisfying the growth condition (2.14), Corollary 2.3 (i) shows (with the choice $a=1$ and $y_1=0$) that one can insert the Gaussian $e^{-\varepsilon (y-y_0)^2}$ and view the integral (2.1) as the limit

$$\begin{aligned} \lim \limits _{\varepsilon \rightarrow 0^+}\int _{\mathbb {R}}e^{-\varepsilon (y-y_0)^2}e^{iy^2}f(y)\mathrm{d}y. \end{aligned}$$

Under the stronger assumption (2.16) (which, in particular, implies that *f* is bounded on the real line) such a regularisation is not necessary and according to Corollary 2.3 (ii) one can regard the integral (2.1) as the limit

$$\begin{aligned} \lim \limits _{R_1,R_2\rightarrow \infty }\int _{-R_1}^{R_2}e^{iy^2}f(y)\mathrm{d}y. \end{aligned}$$

However, under both assumptions (2.14) and (2.16), one has the absolutely convergent representation

$$\begin{aligned} e^{i\alpha }\int _{\mathbb {R}}e^{i(ye^{i\alpha })^2}f(ye^{i\alpha })\mathrm{d}y. \end{aligned}$$

## Green’s functions and solutions of the Schrödinger equation

The main goal of this section is to treat the Cauchy problem (1.1) for the time-dependent one-dimensional Schrödinger equation and the integral representation (1.4) of the solution in a mathematical rigorous framework. In particular, we provide a class of Green’s functions and initial conditions such that (1.4) can be interpreted as a Fresnel integral.

For our purposes, it is convenient to view solutions (and their derivatives) in the context of absolutely continuous functions. Recall that for an interval $I\subseteq {\mathbb {R}}$, a function $f:I\rightarrow {\mathbb {C}}$ is said to be *absolutely continuous*, if there exists some $g\in L^{1}_{\text {loc}}(I)$, such that

3.1 $$\begin{aligned} f(y)-f(x)=\int _x^y g(s)\mathrm{d}s,\qquad x,y\in I. \end{aligned}$$

The linear space of absolutely continuous functions on *I* will be denoted by ${\text {AC}}(I)$. Observe that $f\in {\text {AC}}(I)$ is differentiable almost everywhere and its derivative $f'$ coincides with *g* in (3.1) almost everywhere. For $T\in (0,\infty ]$ we shall work with the space

3.2 $$\begin{aligned}&{\text{ AC }}_{1,2}((0,T)\times {\mathbb {R}})\nonumber \\&\quad :=\left\{ \Psi :(0,T)\times {\mathbb {R}}\rightarrow {\mathbb {C}} \,\bigg |\, \begin{array}{l} \Psi (\,\cdot \,,x)\in {\text{ AC }}((0,T)) \text{ for } \text{ all } x\in {\mathbb {R}} \\ \Psi (t,\,\cdot \,),\frac{\partial }{\partial x}\Psi (t,\,\cdot \,)\in {\text{ AC }}({\mathbb {R}}) \text{ for } \text{ all } t\in (0,T) \end{array}\right\} .\nonumber \\ \end{aligned}$$

Let $V:(0,T)\times {\mathbb {R}}\rightarrow {\mathbb {C}}$ be some potential and let $F:{\mathbb {R}}\rightarrow {\mathbb {C}}$ be some initial condition. We call a function $\Psi \in {\text {AC}}_{1,2}((0,T)\times {\mathbb {R}})$ a *solution* of the time-dependent Schrödinger equation, if it satisfies

3.3a $$\begin{aligned} i\frac{\partial }{\partial t}\Psi (t,x)&=\Big (-\frac{\partial ^2}{\partial x^2}+V(t,x)\Big )\Psi (t,x),&\text {for a.e. }t\in (0,T),\,x\in {\mathbb {R}}, \end{aligned}$$

3.3b $$\begin{aligned} \lim \limits _{t\rightarrow 0^+}\Psi (t,x)&=F(x),&x\in {\mathbb {R}}. \end{aligned}$$

The corresponding *Green’s function* is a function $G:(0,T)\times {\mathbb {R}}\times {\mathbb {R}}\rightarrow {\mathbb {C}}$ (which depends on the potential *V*, but is independent of the initial condition *F*), such that $\Psi$ admits the representation

3.4 $$\begin{aligned} \Psi (t,x)=\int _{\mathbb {R}}G(t,x,y)F(y)\mathrm{d}y,\qquad t\in (0,T),\,x\in {\mathbb {R}}. \end{aligned}$$

Next, we collect a set of assumptions on the Green’s function *G* which ensure that the wave function (3.4) is well-defined and a solution of the Cauchy problem (3.3). The precise formulation of this statement, and also the set of allowed initial conditions, is given in Theorem 3.4.

### Assumption 3.1

Let $T\in (0,\infty ]$ and consider a function

3.5 $$\begin{aligned} G:(0,T)\times {\mathbb {R}}\times {\mathbb {R}}\rightarrow {\mathbb {C}}. \end{aligned}$$

Let $\Omega \subseteq {\mathbb {C}}$ be an open set, which contains the double sector $S_\alpha$ defined in (2.13) for some $\alpha \in (0,\frac{\pi }{2})$ and suppose that *G* admits a continuation to a function

$$G:(0,T)\times {\mathbb {R}}\times \Omega \rightarrow {\mathbb {C}},$$

such that $z\mapsto G(t,x,z)$ is holomorphic on $\Omega$ for every fixed $t\in (0,T)$, $x\in {\mathbb {R}}$. It will be assumed that the following properties (i)–(iv) hold.

(i): For every fixed $z\in S_\alpha$ the function $G(\,\cdot \,,\,\cdot \,,z)\in {\text {AC}}_{1,2}((0,T)\times {\mathbb {R}})$ is a solution of the time-dependent Schrödinger equation
  3.6 $$\begin{aligned} i\frac{\partial }{\partial t}G(t,x,z)=\Big (-\frac{\partial ^2}{\partial x^2}+V(t,x)\Big )G(t,x,z)\quad \text {for a.e. }t\in (0,T),\,x\in {\mathbb {R}}.\nonumber \\ \end{aligned}$$
(ii): There exists $a\in {\text {AC}}((0,T))$ with $a(t)>0$ and $\lim _{t\rightarrow 0^+}a(t)=\infty$, such that the function ${\widetilde{G}}$ in the decomposition
  3.7 $$\begin{aligned} G(t,x,z)=e^{ia(t)(z-x)^2}{\widetilde{G}}(t,x,z),\qquad t\in (0,T),\,x\in {\mathbb {R}},\,z\in \Omega , \end{aligned}$$
  satisfies
  3.8 $$\begin{aligned} |{\widetilde{G}}(t,x,z)|\le A_0(t,x)e^{B_0(t,x)|z|},\qquad t\in (0,T),\,x\in {\mathbb {R}},\,z\in \Omega . \end{aligned}$$
  Here, $A_0,B_0:(0,T)\times {\mathbb {R}}\rightarrow [0,\infty )$ are nonnegative continuous functions, such that
  3.9 $$\begin{aligned} \frac{A_0}{\sqrt{a}}\text { and }B_0\text { extend continuously to }[0,T)\times {\mathbb {R}}. \end{aligned}$$
(iii): For all $x\in {\mathbb {R}}$ and $z\in \Omega$, one has
  3.10 $$\begin{aligned} \lim \limits _{t\rightarrow 0^+}\frac{{\widetilde{G}}(t,x,z)}{\sqrt{a(t)}}=\frac{1}{\sqrt{i\pi }}. \end{aligned}$$
(iv): There exist a nonnegative function $A_1\in L^1_{\text {loc}}((0,T)\times {\mathbb {R}})$ and a nonnegative continuous function $B_1:(0,T)\times {\mathbb {R}}\rightarrow [0,\infty )$, such that for every fixed $t\in (0,T)$ the spatial derivatives of ${{\widetilde{G}}}$ and for every fixed $x\in {\mathbb {R}}$ the time derivative of ${\widetilde{G}}$ are exponentially bounded, that is, the bounds
  3.11 $$\begin{aligned} \Big |\frac{\partial }{\partial x}{\widetilde{G}}(t,x,z)\Big |,\,\Big |\frac{\partial ^2}{\partial x^2}{\widetilde{G}}(t,x,z)\Big |,\,\Big |\frac{\partial }{\partial t}{\widetilde{G}}(t,x,z)\Big |\le A_1(t,x)e^{B_1(t,x)|z|} \end{aligned}$$
  hold for all $z\in S_\alpha$.

We briefly comment on some of the conditions in Assumption 3.1 and refer the reader to Sect. 5 for explicit examples of Green’s functions that satisfy Assumption 3.1. In this context, we also point out that it is of particular interest to derive conditions on the potential *V* such that the corresponding Green’s function satisfies Assumption 3.1.

### Remark 3.2

The assumption on the holomorphy of *G* on $\Omega$ in the *z*-variable is needed to apply the Fresnel integral technique in Corollary 2.3. In explicit examples and applications, one typically starts with a Green’s function as in (3.5) and verifies that it admits a holomorphic continuation to $G:(0,T)\times {\mathbb {R}}\times \Omega \rightarrow {\mathbb {C}}$. The crucial assumption that allows to apply the Fresnel integral method is the decomposition (3.7), where the exponential $e^{ia(t)(z-x)^2}$ (quadratic in *z*) is separated from the remainder ${\widetilde{G}}$, which admits the (at most linear) exponential growth (3.8). The rotation of the integration path in (2.15) from the real line into the complex plane, turns the factor $e^{ia(t)(z-x)^2}$ into a Gaussian which then dominates the linear exponential growth of ${\widetilde{G}}$ in the integral (3.4).

### Remark 3.3

We do not explicitly require the Green’s function to converge to the delta distribution

3.12 $$\begin{aligned} \lim _{t\rightarrow 0^+}G(t,x,y)=\delta (x-y). \end{aligned}$$

In our situation, the counterpart of this standard assumption is the limit condition (3.10), which is the key ingredient to ensure the initial value (3.3b) of the wave function. For us, the limit condition (3.10) is convenient, since in examples it is often easier to check than (3.12).

The next theorem is the main result in the abstract part of this paper. It will be shown that under Assumption 3.1, the integral (3.4) is meaningful as a Fresnel integral and that the resulting function $\Psi$ is a solution of the time-dependent Schrödinger equation (3.3).

### Theorem 3.4

Let $G:(0,T)\times {\mathbb {R}}\times {\mathbb {R}}\rightarrow {\mathbb {C}}$ be as in Assumption 3.1. Furthermore, let $F:{\mathbb {R}}\rightarrow {\mathbb {C}}$ be some initial condition, which admits a holomorphic continuation to the complex domain $\Omega$ from Assumption 3.1, and satisfies the estimate

3.13 $$\begin{aligned} |F(z)|\le Ae^{B|z|},\qquad z\in \Omega , \end{aligned}$$

for some $A,B\ge 0$. Then, the wave function

3.14 $$\begin{aligned} \Psi (t,x):=\lim \limits _{\varepsilon \rightarrow 0^+}\int _{\mathbb {R}}e^{-\varepsilon y^2}G(t,x,y)F(y)\mathrm{d}y,\quad t\in (0,T),\,x\in {\mathbb {R}}, \end{aligned}$$

exists and $\Psi \in {\text {AC}}_{1,2}((0,T)\times {\mathbb {R}})$ is a solution of the Cauchy problem

3.15a $$\begin{aligned} i\frac{\partial }{\partial t}\Psi (t,x)&=\Big (-\frac{\partial ^2}{\partial x^2}+V(t,x)\Big )\Psi (t,x),&\text {for a.e. }t\in (0,T),\,x\in {\mathbb {R}}, \end{aligned}$$

3.15b $$\begin{aligned} \lim \limits _{t\rightarrow 0^+}\Psi (t,x)&=F(x),&x\in {\mathbb {R}}. \end{aligned}$$

### Remark 3.5

If we replace the growth condition (3.8) in Assumption 3.1 (ii) by the stronger condition

3.16 $$\begin{aligned} |{\widetilde{G}}(t,x,z)|\le A_0(t,x)e^{B_0(t,x)|{\text {Im}}(z)|},\qquad t\in (0,T),\,x\in {\mathbb {R}},\,z\in \Omega , \end{aligned}$$

and also replace (3.13) by the stronger condition

3.17 $$\begin{aligned} |F(z)|\le Ae^{B|{\text {Im}}(z)|},\qquad z\in \Omega , \end{aligned}$$

then it follows from Corollary 2.3 (ii) that the wave function (3.14) can be written in the equivalent form

$$\begin{aligned} \Psi (t,x)=\lim \limits _{R_1,R_2\rightarrow \infty }\int _{-R_1}^{R_2}G(t,x,y)F(y)\mathrm{d}y,\quad t\in (0,T),\,x\in {\mathbb {R}}. \end{aligned}$$

We point out that the stronger growth condition (3.16) on the Green’s function is satisfied in all applications in Sect. 5. However, the growth condition (3.17) for the initial condition *F* is rather restrictive and it is desirable to allow also initial conditions that may be unbounded on the real line. See for example the type of initial conditions which arise naturally for the supershift property in Theorem 4.5.

### Proof of Theorem 3.4

*Step 1.* In the first step, we apply Corollary 2.3, to show that the expression (3.14) for the wave function is meaningful. For this, we fix $t\in (0,T)$, $x\in {\mathbb {R}}$ and use the estimates (3.8) and (3.13) to get

3.18 $$\begin{aligned} |{\widetilde{G}}(t,x,z)F(z)|\le AA_0(t,x)e^{(B+B_0(t,x))|z|},\qquad z\in S_\alpha . \end{aligned}$$

Hence, due to the decomposition (3.7), the assumptions of Corollary 2.3 are satisfied, which means that the wave function (3.14) exists and admits the absolutely convergent representation

3.19 $$\begin{aligned} \Psi (t,x)&=\lim \limits _{\varepsilon \rightarrow 0^+}\int _{\mathbb {R}}e^{-\varepsilon y^2}e^{ia(t)(y-x)^2}{\widetilde{G}}(t,x,y)F(y)\mathrm{d}y \nonumber \\&=e^{i\alpha }\int _{\mathbb {R}}e^{ia(t)(ye^{i\alpha }-x)^2}{\widetilde{G}}(t,x,ye^{i\alpha })F(ye^{i\alpha })\mathrm{d}y \nonumber \\&=e^{i\alpha }\int _{\mathbb {R}}G(t,x,ye^{i\alpha })F(ye^{i\alpha })\mathrm{d}y. \end{aligned}$$

*Step 2.* We show that the function $\Psi$ in (3.14) is a solution of the Schrödinger equation (3.15a). Roughly speaking, since *G* is already a solution of (3.6) by Assumption 3.1 (i), one needs to check that wave function (3.19) belongs to the space ${\text {AC}}_{1,2}((0,T)\times {\mathbb {R}})$ and that the derivatives can be carried inside the integral.

Note first, that $G(\,\cdot \,,x,z)\in {\text {AC}}((0,T))$ for every $x\in {\mathbb {R}}$, $z\in \Omega$ by Assumption 3.1 (i) and hence for any $t_0\in (0,T)$, we have

$$\begin{aligned} G(t,x,z)=G(t_0,x,z)+\int _{t_0}^t\frac{\partial }{\partial \tau }G(\tau ,x,z)\mathrm{{d}}\tau ,\quad t\in (0,T),\,x\in {\mathbb {R}},\,z\in S_\alpha . \end{aligned}$$

This leads to the following integral representation of the wave function (3.19)

3.20 $$\begin{aligned} \Psi (t,x)=\Psi (t_0,x)+e^{i\alpha }\int _{\mathbb {R}}\int _{t_0}^t\frac{\partial }{\partial \tau }G(\tau ,x,ye^{i\alpha })\mathrm{{d}}\tau F(ye^{i\alpha })\mathrm{d}y. \end{aligned}$$

Using the decomposition (3.7), we can write the derivative as

$$\begin{aligned} \frac{\partial }{\partial \tau }G(\tau ,x,ye^{i\alpha })=\Big (ia'(\tau )(ye^{i\alpha }-x)^2{\widetilde{G}}(\tau ,x,ye^{i\alpha })+\frac{\partial }{\partial \tau }{\widetilde{G}}(\tau ,x,ye^{i\alpha })\Big )e^{ia(\tau )(ye^{i\alpha }-x)^2}. \end{aligned}$$

Then, we use (3.8), (3.11) and (3.13) to estimate the integrand in (3.20) by

$$\begin{aligned} \begin{aligned}&\Big |\frac{\partial }{\partial \tau }G(\tau ,x,ye^{i\alpha })F(ye^{i\alpha })\Big | \\&\quad \le A\Big (A_0(\tau ,x)|a'(\tau )||ye^{i\alpha }-x|^2e^{B_0(\tau ,x)|y|}+A_1(\tau ,x)e^{B_1(\tau ,x)|y|}\Big ) \\&\qquad \cdot e^{(B+2|x|a(\tau )\sin (\alpha ))|y|}e^{-a(\tau )\sin (2\alpha )y^2}. \end{aligned} \end{aligned}$$

Since the functions $A_0(\,\cdot \,,x)$, $B_0(\,\cdot \,,x)$, $B_1(\,\cdot \,,x)$, $a(\,\cdot \,)$ are continuous and $a'(\,\cdot \,),A_1(\,\cdot \,,x)\in L^{1}_{\text {loc}}((0,T))$ by Assumption 3.1, it follows that the integrand in (3.20) is integrable on $[t_0,t]$. Moreover, the factor $e^{-a(\tau )\sin (2\alpha )y^2}$ also implies integrability with respect to $y\in {\mathbb {R}}$, and hence the integrand is absolutely integrable on $[t_0,t]\times {\mathbb {R}}$. Therefore, the order of integration in (3.20) can be interchanged and we obtain

$$\begin{aligned} \Psi (t,x)=\Psi (t_0,x)+e^{i\alpha }\int _{t_0}^t\int _{\mathbb {R}}\frac{\partial }{\partial \tau }G(\tau ,x,ye^{i\alpha })F(ye^{i\alpha })\mathrm{d}y\mathrm{{d}}\tau . \end{aligned}$$

In particular, this shows $\Psi (\,\cdot \,,x)\in {\text {AC}}((0,T))$ and the derivative with respect to *t* exists almost everywhere and is given by

$$\begin{aligned} \frac{\partial }{\partial t}\Psi (t,x)=e^{i\alpha }\int _{\mathbb {R}}\frac{\partial }{\partial t}G(t,x,ye^{i\alpha })F(ye^{i\alpha })\mathrm{d}y. \end{aligned}$$

Using the same argument, also $\Psi (t,\,\cdot \,)$ and $\frac{\partial }{\partial x}\Psi (t,\,\cdot \,)$ are absolutely continuous, with spatial derivatives almost everywhere given by

$$\begin{aligned} \frac{\partial }{\partial x}\Psi (t,x)&=e^{i\alpha }\int _{\mathbb {R}}\frac{\partial }{\partial x}G(t,x,ye^{i\alpha })F(ye^{i\alpha })\mathrm{d}y, \\ \frac{\partial ^2}{\partial x^2}\Psi (t,x)&=e^{i\alpha }\int _{\mathbb {R}}\frac{\partial ^2}{\partial x^2}G(t,x,ye^{i\alpha })F(ye^{i\alpha })\mathrm{d}y. \end{aligned}$$

This means $\Psi \in {\text {AC}}_{1,2}((0,T)\times {\mathbb {R}})$ and from (3.6), we conclude that the Schrödinger equation (3.15a) is satisfied for a.e. $t\in (0,T)$, $x\in {\mathbb {R}}$.

*Step 3.* Now, we verify the initial condition (3.15b). For this, we fix $x\in {\mathbb {R}}$ and split up the integral (3.14) as

$$\begin{aligned} \Psi (t,x)= \Psi _1(t,x)+\Psi _0(t,x)+\Psi _2(t,x), \end{aligned}$$

where we have set

$$\begin{aligned} \begin{aligned} \Psi _1(t,x)&=\lim \limits _{\varepsilon \rightarrow 0^+}\int _{-\infty }^{y_1}e^{-\varepsilon y^2}G(t,x,y)F(y)\mathrm{d}y, \\ \Psi _0(t,x)&=\lim \limits _{\varepsilon \rightarrow 0^+}\int _{y_1}^{y_2}e^{-\varepsilon y^2}G(t,x,y)F(y)\mathrm{d}y, \\ \Psi _2(t,x)&=\lim \limits _{\varepsilon \rightarrow 0^+}\int _{y_2}^\infty e^{-\varepsilon y^2}G(t,x,y)F(y)\mathrm{d}y, \end{aligned} \end{aligned}$$

and $y_1<0$ and $y_2>0$ are chosen such that $x\in (y_1,y_2)$. Starting with $\Psi _2$, we use the fact that the shifted sector $y_2+S_\alpha ^+$ is contained in $S_\alpha ^+$. A similar estimate as in (3.18) shows that we may apply Corollary 2.2 (i) to ${\widetilde{G}}(t,x,y+y_2)F(y+y_2)$ and find

$$\begin{aligned} \Psi _2(t,x)&=\lim \limits _{\varepsilon \rightarrow 0^+}\int _{y_2}^\infty e^{-\varepsilon y^2}e^{ia(t)(y-x)^2}{\widetilde{G}}(t,x,y)F(y)\mathrm{d}y \\&=\lim \limits _{\varepsilon \rightarrow 0^+}\int _0^\infty e^{-\varepsilon (y+y_2)^2}e^{ia(t)(y+y_2-x)^2}{\widetilde{G}}(t,x,y+y_2)F(y+y_2)\mathrm{d}y \\&=e^{i\alpha }\int _0^\infty e^{ia(t)(ye^{i\alpha }+y_2-x)^2}{\widetilde{G}}(t,x,ye^{i\alpha }+y_2)F(ye^{i\alpha }+y_2)\mathrm{d}y \\&=e^{i\alpha }\int _0^\infty G(t,x,ye^{i\alpha }+y_2)F(ye^{i\alpha }+y_2)\mathrm{d}y. \end{aligned}$$

In this form, we can estimate $\Psi _2(t,x)$ as

3.21 $$\begin{aligned} |\Psi _2(t,x)|&\le \int _0^\infty \big |e^{ia(t)(ye^{i\alpha }+y_2-x)^2}{\widetilde{G}}(t,x,ye^{i\alpha }+y_2)F(ye^{i\alpha }+y_2)\big |\mathrm{d}y \nonumber \\&\le AA_0(t,x)\int _0^\infty e^{-a(t)(y^2\sin (2\alpha )+2(y_2-x)y\sin (\alpha ))}e^{(B+B_0(t,x))|ye^{i\alpha }+y_2|}\mathrm{d}y \nonumber \\&\le AA_0(t,x)e^{(B+B_0(t,x))y_2}\int _0^\infty e^{-a(t)\sin (2\alpha )y^2}e^{(B+B_0(t,x)-2a(t)(y_2-x)\sin (\alpha ))y}\mathrm{d}y \nonumber \\&=\frac{AA_0(t,x)\sqrt{\pi }}{2\sqrt{a(t)\sin (2\alpha )}}e^{(B+B_0(t,x))y_2}\Lambda \bigg (\frac{\sqrt{a(t)\tan (\alpha )}}{\sqrt{2}}\Big (y_2-x-\frac{B+B_0(t,x)}{2a(t)}\Big )\bigg ), \end{aligned}$$

where, for a shorter notation, we used $\Lambda (\xi ):= e^{\xi ^2}(1-{\text {erf}}(\xi ))$ as a modification of the well-known error function; for the computation of the integral see [4, Lemma 2.1]. According to Assumption 3.1 (ii), we know that $\frac{A_0}{\sqrt{a}}$ and $B_0$ remain finite for $t\rightarrow 0^+$, and also that $a\rightarrow \infty$ for $t\rightarrow 0^+$. Therefore, since $\lim _{\xi \rightarrow \infty }\Lambda (\xi )=0$, see [1, Formula 7.1.23], and $x<y_2$ we conclude

3.22 $$\begin{aligned} \lim \limits _{t\rightarrow 0^+}\Psi _2(t,x)=0. \end{aligned}$$

In the same way, one verifies that

3.23 $$\begin{aligned} \lim \limits _{t\rightarrow 0^+}\Psi _1(t,x)=0. \end{aligned}$$

For the limit of $\Psi _0$ for $t\rightarrow 0^+$, we first note that due to the dominated convergence theorem, we can write

$$\begin{aligned} \Psi _0(t,x)=\int _{y_1}^{y_2}G(t,x,y)F(y)\mathrm{d}y. \end{aligned}$$

Using the derivative $\frac{\mathrm{d}}{\mathrm{{d}}\xi }{\text {erf}}(\xi )=\frac{2}{\sqrt{\pi }}e^{-\xi ^2}$ of the error function together with the decomposition (3.7), we rewrite the integral as

$$\begin{aligned} \Psi _0(t,x)=\frac{\sqrt{\pi }}{2i\sqrt{ia(t)}}\int _{y_1}^{y_2}\bigg (\frac{\partial }{\partial y}{\text {erf}}\big (i\sqrt{ia(t)}(y-x)\big )\bigg ){\widetilde{G}}(t,x,y)F(y)\mathrm{d}y. \end{aligned}$$

Applying integration by parts then leads to the four terms

3.24 $$\begin{aligned} \begin{aligned} \Psi _0(t,x)=\frac{\sqrt{i\pi }}{2}\bigg (&-{\text{ erf }}\big (i\sqrt{ia(t)}(y_2-x)\big )\frac{{\widetilde{G}}(t,x,y_2)}{\sqrt{a(t)}}F(y_2) \\ {}&+{\text{ erf }}\big (i\sqrt{ia(t)}(y_1-x)\big )\frac{{\widetilde{G}}(t,x,y_1)}{\sqrt{a(t)}}F(y_1) \\ {}&+\int _{y_1}^{y_2}{\text{ erf }}\big (i\sqrt{ia(t)}(y-x)\big )\frac{\frac{\partial }{\partial y}{\widetilde{G}}(t,x,y)}{\sqrt{a(t)}}F(y)\mathrm {d}y \\ {}&+\int _{y_1}^{y_2}{\text{ erf }}\big (i\sqrt{ia(t)}(y-x)\big )\frac{{\widetilde{G}}(t,x,y)}{\sqrt{a(t)}}F'(y)\mathrm {d}y\bigg ). \end{aligned} \end{aligned}$$

Due to the limit

$$\begin{aligned} \lim \limits _{a\rightarrow \infty }{\text {erf}}\big (i\sqrt{ia}\,\xi \big )={\text {sgn}}(-\xi )=\left\{ \begin{array}{ll} 1, &{} \text {if }\xi <0, \\ -1, &{} \text {if }\xi >0, \end{array}\right. \end{aligned}$$

of the error function [1, Formula 7.1.23], as well as the limit (3.10), we find for the first two terms in (3.24)

$$\begin{aligned} \lim \limits _{t\rightarrow 0^+}{\text {erf}}\big (i\sqrt{ia(t)}(y_2-x)\big )\frac{{\widetilde{G}}(t,x,y_2)}{\sqrt{a(t)}}F(y_2)&=-\frac{F(y_2)}{\sqrt{i\pi }}, \\ \lim \limits _{t\rightarrow 0^+}{\text {erf}}\big (i\sqrt{ia(t)}(y_1-x)\big )\frac{{\widetilde{G}}(t,x,y_1)}{\sqrt{a(t)}}F(y_1)&=\frac{F(y_1)}{\sqrt{i\pi }}. \end{aligned}$$

Consider now the fourth term in (3.24). Due to the estimate (3.8), we have

$$\begin{aligned} \frac{|{\widetilde{G}}(t,x,y)|}{\sqrt{a(t)}}\le \frac{A_0(t,x)}{\sqrt{a(t)}}e^{B_0(t,x)|y|},\qquad y\in [y_1,y_2], \end{aligned}$$

which is uniformly bounded for $t\rightarrow 0^+$ by (3.9). Since also the error function $\xi \mapsto {\text {erf}}(i\sqrt{i}\,\xi )$ is bounded on ${\mathbb {R}}$, there exists a *t*-uniform majorant, and hence, the limit can be carried inside the integral. This gives

$$\begin{aligned} \lim \limits _{t\rightarrow 0^+}\int _{y_1}^{y_2}{\text {erf}}\big (i\sqrt{ia(t)}(y-x)\big )\frac{{\widetilde{G}}(t,x,y)}{\sqrt{a(t)}}F'(y)\mathrm{d}y&=\frac{1}{\sqrt{i\pi }}\int _{y_1}^{y_2}{\text {sgn}}(x-y)F'(y)\mathrm{d}y \\&=\frac{2F(x)-F(y_1)-F(y_2)}{\sqrt{i\pi }}. \end{aligned}$$

For the third term in (3.24), we note that the compact interval $[y_1,y_2]$ is contained in the open set $\Omega$. Hence, there exists some $r>0$ such that for every $y\in [y_1,y_2]$ the closed ball $B_r(y)$ is contained in $\Omega$. The Cauchy integral formula then gives

3.25 $$\begin{aligned} \frac{\partial }{\partial y}{\widetilde{G}}(t,x,y)=\frac{1}{2\pi i}\int _{\partial B_r(y)}\frac{{\widetilde{G}}(t,x,z)}{(z-y)^2}\mathrm{d}z=\frac{1}{2\pi r}\int _0^{2\pi }{\widetilde{G}}(t,x,y+re^{i\varphi })e^{-i\varphi }\mathrm{{d}}\varphi .\nonumber \\ \end{aligned}$$

Due to (3.8), we can estimate the integrand as

3.26 $$\begin{aligned} \frac{|{\widetilde{G}}(t,x,y+re^{i\varphi })|}{\sqrt{a(t)}}\le \frac{A_0(t,x)}{\sqrt{a(t)}}e^{B_0(t,x)|y+re^{i\varphi }|}\le \frac{A_0(t,x)}{\sqrt{a(t)}}e^{B_0(t,x)(|y|+r)},\qquad \end{aligned}$$

and from (3.9), we see that it admits an *t*-independent upper bound near $t=0^+$. Hence, we are allowed to carry the limit inside the integral and get

3.27 $$\begin{aligned} \lim \limits _{t\rightarrow 0^+}\frac{\frac{\partial }{\partial y}{\widetilde{G}}(t,x,y)}{\sqrt{a(t)}}= & {} \frac{1}{2\pi r}\int _0^{2\pi }\lim \limits _{t\rightarrow 0^+}\frac{{\widetilde{G}}(t,x,y+re^{i\varphi })}{\sqrt{a(t)}}e^{-i\varphi }\mathrm{{d}}\varphi \nonumber \\= & {} \frac{1}{2\pi r\sqrt{i\pi }}\int _0^{2\pi }e^{-i\varphi }\mathrm{{d}}\varphi =0. \end{aligned}$$

Moreover, by the representation (3.25) and the estimate (3.26), we get

$$\begin{aligned} \frac{|\frac{\partial }{\partial y}{\widetilde{G}}(t,x,y)|}{\sqrt{a(t)}}\le \frac{A_0(t,x)}{r\sqrt{a(t)}}e^{B_0(t,x)(|y|+r)},\qquad y\in [y_1,y_2]. \end{aligned}$$

Again, by (3.9), we obtain an integrable and *t*-independent upper bound of the third term in (3.24). Hence, we are allowed to carry the limit $t\rightarrow 0^+$ inside the integral, which vanishes according to (3.27). Altogether this shows that (3.24) converges to

$$\begin{aligned} \lim \limits _{t\rightarrow 0^+}\Psi _0(t,x)=\frac{1}{2}\Big (F(y_2)+F(y_1)+2F(x)-F(y_1)-F(y_2)\Big )=F(x). \end{aligned}$$

Together with (3.22) and (3.23), this finally proves the initial value (3.15b). $\square$

In the next theorem, we show that the solution $\Psi$ of the Schrödinger equation depends continuously on the initial condition. Note that the assumed convergence (3.28) of the initial condition is stronger than the resulting uniform convergence on compact sets (3.29) at times $t>0$. However, the stronger convergence (3.28) is justified, since this is the standard type of convergence in which superoscillations are normally treated, see for example (4.6). For convenience, we use the notation $\Psi (t,x;F)$ to emphasize the initial condition.

### Theorem 3.6

Let $G:(0,T)\times {\mathbb {R}}\times {\mathbb {R}}\rightarrow {\mathbb {C}}$ be as in Assumption 3.1. Moreover, let $F,(F_n)_n:{\mathbb {R}}\rightarrow {\mathbb {C}}$ be initial conditions which admit holomorphic extensions to $\Omega$ and satisfy the growth condition (3.13) for some $A,B,(A_n)_n,(B_n)_n\ge 0$. If the sequence $(F_n)_n$ converges as

3.28 $$\begin{aligned} \lim \limits _{n\rightarrow \infty }\sup \limits _{z\in \Omega }|F(z)-F_n(z)|e^{-C|z|}=0 \end{aligned}$$

for some $C\ge 0$, then also the corresponding wave functions converge as

3.29 $$\begin{aligned} \lim \limits _{n\rightarrow \infty }\Psi (t,x;F_n)=\Psi (t,x;F) \end{aligned}$$

uniformly on compact subsets of $[0,T)\times {\mathbb {R}}$.

### Proof

Since the double sector $S_\alpha$ is contained in $\Omega$, there exists for every $x\in {\mathbb {R}}$ some $\beta (x)\in (0,\alpha ]$, continuously depending on *x*, such that the shifted double sector $x+S_{\beta (x)}$ is contained in $\Omega$. In the same way as in (3.19), we get the representation

3.30 $$\begin{aligned} \Psi (t,x;F)=e^{i\beta (x)}\int _{\mathbb {R}}G(t,x,x+ye^{i\beta (x)})F(x+ye^{i\beta (x)}),\qquad t\in (0,T),\,x\in {\mathbb {R}}.\nonumber \\ \end{aligned}$$

If we define

$$\begin{aligned} L_n:=\sup _{z\in \Omega }|F(z)-F_n(z)|e^{-C|z|} \end{aligned}$$

from (3.28), we can use (3.7) and (3.8) to estimate

$$\begin{aligned} |\Psi (t,x;F)-\Psi (t,x;F_n)|&\le \int _{\mathbb {R}}\big |G(t,x,x+ye^{i\beta (x)})\big |\big |F(x+ye^{i\beta (x)})-F_n(x+ye^{i\beta (x)})\big |\mathrm{d}y \\&\le L_nA_0(t,x)\int _{\mathbb {R}}e^{-a(t)\sin (2\beta (x))y^2}e^{(C+B_0(t,x))|x+ye^{i\beta (x)}|}\mathrm{d}y \\&\le L_nA_0(t,x)e^{(C+B_0(t,x))|x|}\int _{\mathbb {R}}e^{-a(t)\sin (2\beta (x))y^2+(C+B_0(t,x))|y|}\mathrm{d}y \\&=\frac{L_nA_0(t,x)\sqrt{\pi }}{\sqrt{a(t)\sin (2\beta (x))}}e^{(C+B_0(t,x))|x|}\Lambda \bigg (-\frac{C+B_0(t,x)}{2\sqrt{a(t)\sin (2\beta (x))}}\bigg ), \end{aligned}$$

where the analytic value of the last integral is similar to the one in (3.21). Since $L_n\overset{n\rightarrow \infty }{\longrightarrow }0$ by (3.28) and the right-hand side is continuous in *t* and *x* by (3.9) and the continuity of $\beta$, it follows that the convergence (3.29) is uniform on compact subsets of $[0,T)\times {\mathbb {R}}$. $\square$

## Supershifts and superoscillations

The aim of this section is to investigate the time evolution of superoscillations and the supershift property of the solution $\Psi$ of the one-dimensional Schrödinger equation (3.3). As already mentioned in the introduction, the main novelty of our unified approach, with respect to the existing literature, is, that we are able to consider potentials, where the explicit form of the Green’s function is not known. Instead, our results are only based on the regularity and growth conditions on the Green’s functions, see Assumption 3.1.

We start with the abstract definition of a supershift and explain its usefulness and meaning afterwards.

### Definition 4.1

(*Supershift*). Let ${\mathcal {O}},{\mathcal {U}}\subseteq {\mathbb {C}}$ such that ${\mathcal {U}}\subsetneqq {\mathcal {O}}$. Let *X* be a metric space and consider a family

4.1 $$\begin{aligned} \varphi _\kappa :X\rightarrow {\mathbb {C}}, \qquad \kappa \in {\mathcal {O}}, \end{aligned}$$

of complex valued functions. We say that a sequence of functions $(\Phi _n)_n$ of the form

4.2 $$\begin{aligned} \Phi _n(s)=\sum \limits _{l=0}^nC_l(n)\varphi _{\kappa _l(n)}(s) ,\qquad s\in X, \end{aligned}$$

with coefficients $C_l(n)\in {\mathbb {C}}$, $\kappa _l(n)\in {\mathcal {U}}$, admits a *supershift*, if there exists some $\kappa \in {\mathcal {O}}{\setminus }{\mathcal {U}}$, such that

4.3 $$\begin{aligned} \lim \limits _{n\rightarrow \infty }\Phi _n(s)=\varphi _\kappa (s),\qquad s\in X, \end{aligned}$$

converges uniformly on compact subsets of *X*.

### Remark 4.2

If the sequence $(\Phi _n)_n$ in (4.2) admits a supershift, then the values of $\varphi _\kappa$ for some $\kappa \in {\mathcal {O}}$, outside the smaller set ${\mathcal {U}}$, can be calculated by only using values $\varphi _{\kappa _l(n)}$ at the points $\kappa _l(n)$ inside ${\mathcal {U}}$. Hence, informally speaking, when considering the mapping $\kappa \mapsto \varphi _\kappa$ in the $\kappa$-variable there is a breeze of analyticity in the air, see also Theorem 4.7 and Corollary 4.8.

Next, we discuss a standard example for the supershift property, see also the example (1.2) in the introduction, as well as Remark 4.4 for the connection to the notion of superoscillations.

### Example 4.3

Let $X={\mathbb {C}}$ and consider for every $\kappa \in {\mathcal {O}}={\mathbb {C}}$ the exponentials

$$\begin{aligned} \varphi _\kappa (z)=e^{i\kappa z},\qquad z\in {\mathbb {C}}. \end{aligned}$$

Let furthermore ${\mathcal {U}}=[-1,1]$ and $\kappa \in {\mathcal {O}}{\setminus } {\mathcal {U}}$ be arbitrary. In [36, Lemma 2.4] it was shown that with the coefficients

4.4 $$\begin{aligned} C_l(n)={n\atopwithdelims ()l}\bigg (\frac{1+\kappa }{2}\bigg )^{n-l}\bigg (\frac{1-\kappa }{2}\bigg )^l \qquad \text {and}\qquad \kappa _l(n)=1-\frac{2l}{n}\in {\mathcal {U}}, \end{aligned}$$

the sequence of functions

$$\begin{aligned} \Phi _n(z)=\sum _{l=0}^nC_l(n)e^{i\kappa _l(n)z},\qquad z\in {\mathbb {C}}, \end{aligned}$$

converges as

4.5 $$\begin{aligned} \lim _{n\rightarrow \infty }\Phi _n(z)=e^{i\kappa z} \end{aligned}$$

uniformly on compact subsets of ${\mathbb {C}}$, that is, for any $\kappa \in {\mathbb {C}}{\setminus }[-1,1]$, the sequence $(\Phi _n)_n$ admits a supershift. Moreover, according to [36, Theorem 2.1], one even has the stronger convergence

4.6 $$\begin{aligned} \lim \limits _{n\rightarrow \infty }\sup \limits _{z\in {\mathbb {C}}}|\Phi _n(z)-e^{i\kappa z}|e^{-C|z|}=0 \end{aligned}$$

for some $C\ge 0$, which agrees with the assumption (3.28) in Theorem 3.6.

In the next remark, we explain the connection between the notion of supershift in Definition 4.1 and the concept of superoscillations, which has attracted a lot of attention in the physical and mathematical literature, see the references mentioned in the introduction. Below, we use the definition of superoscillations from [4, 11].

### Remark 4.4

(Superoscillations). Sequences of the form

4.7 $$\begin{aligned} \Phi _n(x)=\sum _{j=0}^n C_j(n)e^{ik_j(n)x},\qquad x\in {\mathbb {R}}, \end{aligned}$$

with coefficients $C_j(n)\in {\mathbb {C}}$, $k_j(n)\in {\mathbb {R}}$, are often called *generalized Fourier sequences*. Note that with $\varphi _k(x)=e^{ikx}$, $x\in {\mathbb {R}}$, as in Example 4.3, this agrees with the functions in (4.2). The generalized Fourier sequence (4.7) is said to be *superoscillating* if there exists some ${\tilde{k}}\in {\mathbb {R}}$ such that

$$\begin{aligned} k':=\sup _{n\in {\mathbb {N}}_0,\,j\in \{0,\dots ,n\}}|k_j(n)|<|{\tilde{k}}|, \end{aligned}$$

and there exists a compact subset $K\subset {\mathbb {R}}$, called *superoscillation set*, such that

4.8 $$\begin{aligned} \lim \limits _{n\rightarrow \infty }\sup \limits _{x\in K}|\Phi _n(x)-e^{i{\tilde{k}}x}|=0; \end{aligned}$$

cf. [4, Definition 5.1]. Note that the sequence $(\Phi _n)_n$ converges to a plane wave $e^{i{\tilde{k}}x}$ with frequency $|{\tilde{k}}|>k'$, that is, the shift in the *k*-variable is manifested in a shift of the frequencies, which leads to the terminology superoscillations. Observe that superoscillations can be viewed as a special case of the supershift in Definition 4.1 by choosing

$$\begin{aligned} {\mathcal {O}}={\mathbb {R}},\quad {\mathcal {U}}=[-k',k'],\quad \text {and}\quad X=K. \end{aligned}$$

In fact, the supershift property is heavily inspired by the concept of superoscillations and Definition 4.1 is designed in such a way that it applies to the time evolution of solutions of the Schrödinger equation subject to superoscillatory initial data.

The first main result of this section is Theorem 4.5 on the supershift property of the solution of the Schrödinger equation, which can be viewed as a corollary of the continuous dependence result from Theorem 3.6. Roughly speaking, we consider a family of initial conditions that admits a supershift (with respect to a slightly stronger form of convergence as in Definition 4.1) and conclude that the corresponding solutions of the Schrödinger equation admit a similar type of supershift; see also Remark 4.6.

### Theorem 4.5

(Supershift property). Let $G:(0,T)\times {\mathbb {R}}\times {\mathbb {R}}\rightarrow {\mathbb {C}}$ and $\Omega$ be as in Assumption 3.1, let ${\mathcal {O}},{\mathcal {U}}\subseteq {\mathbb {C}}$ with ${\mathcal {U}}\subsetneqq {\mathcal {O}}$, and consider a family of analytic functions $\varphi _\kappa :\Omega \rightarrow {\mathbb {C}}$ with $\kappa \in {\mathcal {O}}$ that satisfy the estimate

4.9 $$\begin{aligned} |\varphi _\kappa (z)|\le A(\kappa )e^{B(\kappa )|z|},\qquad z\in \Omega , \end{aligned}$$

for some $A(\kappa ),B(\kappa )\ge 0$ continuously depending on $\kappa$. If a sequence of initial conditions $(F_n)_n$ of the form

4.10 $$\begin{aligned} F_n(z)=\sum \limits _{l=0}^nC_l(n)\varphi _{\kappa _l(n)}(z),\qquad z\in \Omega , \end{aligned}$$

with coefficients $C_l(n)\in {\mathbb {C}}$, $\kappa _l(n)\in {\mathcal {U}}$, converges as

4.11 $$\begin{aligned} \lim \limits _{n\rightarrow \infty }\sup \limits _{z\in \Omega }|F_n(z)-\varphi _\kappa (z)|e^{-C|z|}=0, \end{aligned}$$

for some $C\ge 0$, to some $\varphi _\kappa$ with $\kappa \in {\mathcal {O}}{\setminus }{\mathcal {U}}$, then the sequence of solutions of the Schrödinger equation converges as

4.12 $$\begin{aligned} \lim \limits _{n\rightarrow \infty }\Psi (t,x;F_n)=\lim \limits _{n\rightarrow \infty }\sum \limits _{l=0}^nC_l(n)\Psi (t,x;\varphi _{\kappa _l(n)})=\Psi (t,x;\varphi _\kappa ) \end{aligned}$$

uniformly on compact subsets of $[0,T)\times {\mathbb {R}}$.

### Proof of Theorem 4.5

The fact that the convergence (4.11) leads to the convergence (4.12), was already proven in Theorem 3.6. Moreover, splitting the solutions $\Psi (t,x;F_n)$ into the given linear combination is allowed due to the linearity of the Schrödinger equation with respect to the initial condition, i.e.

$$\begin{aligned} \Psi (t,x;F_n)=\Psi \Big (t,x;\sum \limits _{l=0}^nC_l(n)\varphi _{\kappa _l(n)}\Big )=\sum \limits _{l=0}^nC_l(n)\Psi (t,x;\varphi _{\kappa _l(n)}). \end{aligned}$$

$$\square $$

### Remark 4.6

Since the convergence (4.11) implies uniform convergence on all compact subsets of $\Omega$, it is clear that the initial conditions $(F_n)_n$ in (4.10) admit the supershift property of Definition 4.1 with respect to the metric space $X=\Omega$. Furthermore, with the metric space $X=[0,T)\times {\mathbb {R}}$ and the functions (4.1) as $\phi _\kappa (t,x):=\Psi (t,x;\varphi _\kappa )$, we are again in the setting of Definition 4.1. The convergence (4.12) shows that the sequence $(\Psi (t,x;F_n))_n$ admits a supershift with respect to the functions $\phi _\kappa$ in the metric space $[0,T)\times {\mathbb {R}}$.

In the next result, we continue the theme of Theorem 4.5 and return to the analyticity issue mentioned in Remark 4.2. In fact, the following Theorem 4.7 shows that analyticity in the $\kappa$-variable in the initial condition implies analyticity in the $\kappa$-variable in the wave function.

### Theorem 4.7

Let $G:(0,T)\times {\mathbb {R}}\times {\mathbb {R}}\rightarrow {\mathbb {C}}$ and $\Omega$ be as in Assumption 3.1, let ${\mathcal {O}}\subseteq {\mathbb {C}}$ be an open set, and consider a family of analytic functions $\varphi _\kappa :\Omega \rightarrow {\mathbb {C}}$ with $\kappa \in {\mathcal {O}}$ that satisfy the estimate

4.13 $$\begin{aligned} |\varphi _\kappa (z)|\le A(\kappa )e^{B(\kappa )|z|},\qquad z\in \Omega , \end{aligned}$$

for some $A(\kappa ),B(\kappa )\ge 0$ continuously depending on $\kappa$, and let $\Psi (t,x;\varphi _\kappa )$ be the corresponding solution of the Schrödinger equation. If for every $z\in \Omega$ the mapping

$$\begin{aligned} {\mathcal {O}}\ni \kappa \mapsto \varphi _\kappa (z) \end{aligned}$$

is holomorphic, then for every fixed $t\in (0,T)$, $x\in {\mathbb {R}}$, the mapping

$$\begin{aligned} {\mathcal {O}}\ni \kappa \mapsto \Psi (t,x;\varphi _\kappa ) \end{aligned}$$

is holomorphic as well.

### Proof

Fix $t\in (0,T)$, $x\in {\mathbb {R}}$. Then for any triangle $\Delta \subseteq {\mathcal {O}}$, we have the path integral

4.14 $$\begin{aligned} \int _\Delta \Psi (t,x;\varphi _\kappa )\mathrm{{d}}\kappa =\int _\Delta e^{i\alpha }\int _{\mathbb {R}}G(t,x,ye^{i\alpha })\varphi _\kappa (ye^{i\alpha })\mathrm{d}y\mathrm{{d}}\kappa , \end{aligned}$$

due to the representation (3.19) of the wave function. Here, $\alpha \in (0,\frac{\pi }{2})$ is the angle of the double sector $S_\alpha$ in Assumption 3.1. In order to interchange the order of integration, we have to prove absolute integrability of the double integral. Firstly, the estimate

4.15 $$\begin{aligned} |G(t,x,ye^{i\alpha })\varphi _\kappa (ye^{i\alpha })|\le A(\kappa )A_0(t,x)e^{-a(t)\sin (2\alpha )y^2}e^{(B(\kappa )+B_0(t,x)+2|x|a(t)\sin (\alpha ))|y|}\nonumber \\ \end{aligned}$$

follows from (3.7), (3.8) and (4.13) and shows that the *y*-integral is absolutely convergent. Moreover, the coefficients $A(\kappa ),B(\kappa )$ are assumed to be continuous and hence this upper bound can be uniformly estimated on the compact triangle $\Delta$. This means that the right hand side of (4.15) can be estimated by some $\kappa$-independent and *y*-integrable upper bound. Hence, the double integral (4.14) is absolutely convergent, and we are allowed to interchange the order of integration and get

$$\begin{aligned} \int _\Delta \Psi (t,x;\varphi _\kappa )\mathrm{{d}}\kappa =e^{i\alpha }\int _{\mathbb {R}}G(t,x,ye^{i\alpha })\int _\Delta \varphi _\kappa (ye^{i\alpha })\mathrm{{d}}\kappa \mathrm{d}y. \end{aligned}$$

Since the mapping $\mathcal O\ni \kappa \mapsto \varphi _\kappa (ye^{i\alpha })$ is holomorphic the path integral along $\Delta$ vanishes, and we get

$$\begin{aligned} \int _\Delta \Psi (t,x;\varphi _\kappa )\mathrm{{d}}\kappa =0. \end{aligned}$$

Due to the Theorem of Morera this implies analyticity of ${\mathcal {O}}\ni \kappa \mapsto \Psi (t,x;\varphi _\kappa )$. $\square$

In order to appreciate our main results, the following corollary shows how the above Theorems 4.5 and 4.7 combine in the special case $\varphi _\kappa (z)=e^{i\kappa z}$ from Example 4.3, which are also the basic functions of superoscillations in Remark 4.4.

### Corollary 4.8

Let $G:(0,T)\times {\mathbb {R}}\times {\mathbb {R}}\rightarrow {\mathbb {C}}$ be as in Assumption 3.1 and consider the exponentials $\varphi _\kappa (z)=e^{i\kappa z}$, $\kappa ,z\in {\mathbb {C}}$, as in Example 4.3. If for any $\kappa \in {\mathbb {C}}{\setminus }[-1,1]$ we choose the coefficients $C_l(n)$ and $\kappa _l(n)$ as in (4.4), then the sequence of solutions $(\Psi (t,x;F_n))_n$ of the Schrödinger equation with initial condition

4.16 $$\begin{aligned} F_n(z)=\sum \limits _{k=0}^nC_l(n)e^{i\kappa _l(n)z},\qquad z\in {\mathbb {C}}, \end{aligned}$$

converges as

4.17 $$\begin{aligned} \lim \limits _{n\rightarrow \infty }\Psi (t,x;F_n)=\lim \limits _{n\rightarrow \infty }\sum \limits _{l=0}^nC_l(n)\Psi (t,x;e^{i\kappa _l(n)\,\cdot \,})=\Psi (t,x;e^{i\kappa \,\cdot \,}) \end{aligned}$$

uniformly on compact subsets of $[0,T)\times {\mathbb {R}}$, that is, $(\Psi (t,x;F_n))_n$ admits a supershift. Moreover, for every fixed $t\in (0,T)$, $x\in {\mathbb {R}}$, the mapping

$$\begin{aligned} {\mathbb {C}}\ni \kappa \mapsto \Psi (t,x;e^{i\kappa \,\cdot \,}) \end{aligned}$$

is analytic.

### Proof

According to (4.6), the initial conditions $(F_n)_n$ converge as

$$\begin{aligned} \lim \limits _{n\rightarrow \infty }\sup \limits _{z\in {\mathbb {C}}}|F_n(z)-e^{i\kappa z}|e^{-C|z|}=0, \end{aligned}$$

for some $C\ge 0$. Hence, it follows from Theorem 4.5 that the sequence of solutions $(\Psi (t,x;F_n))_n$ converges as (4.17) uniformly on compact subsets of $[0,T)\times {\mathbb {R}}$. Moreover, since the mapping ${\mathbb {C}}\ni \kappa \mapsto e^{i\kappa z}$ is analytic for every fixed $z\in {\mathbb {C}}$, it follows from Theorem 4.7 that also the mapping

$$\begin{aligned} {\mathbb {C}}\ni \kappa \mapsto \Psi (t,x;e^{i\kappa \,\cdot \,}) \end{aligned}$$

is analytic for every fixed $t\in (0,T)$, $x\in {\mathbb {R}}$. $\square$

## Some applications of the main results

We are now in the position to apply the results of the previous sections to the time-dependent one-dimensional Schrödinger equation with specific potentials. Here, we consider the case of the free particle, the time-dependent uniform electric field, the time-dependent harmonic oscillator, and the Pöschl–Teller potential. In particular, we formulate a variant of Theorem 4.5. The proof of Theorem 5.1 is direct; in fact, for each potential, we derive the Green’s function and verify Assumption 3.1. From our discussion below, it is also immediate that for each of the following potentials (I)–(IV), the representation of the wave function in Theorem 3.4, the continuous dependency on the initial value in Theorem 3.6, and the analyticity property in Theorem 4.7 hold.

We also refer the reader to [8] for the free particle, [12] for the constant electric field and [16, 32, 36] for the harmonic oscillator that are included in our general setting, while the Pöschl–Teller potential is treated here for the first time. The difference with respect to the previous literature is that the initial condition considered here is not necessarily a superoscillatory function but a supershift.

### Theorem 5.1

Consider the time-dependent Schrödinger equation

5.1a $$\begin{aligned} i\frac{\partial }{\partial t}\Psi (t,x)&=\Big (-\frac{\partial ^2}{\partial x^2}+V(t,x)\Big )\Psi (t,x),&\text {for a.e. }t\in (0,T),\,x\in {\mathbb {R}}, \end{aligned}$$

5.1b $$\begin{aligned} \lim \limits _{t\rightarrow 0^+}\Psi (t,x)&=F_n(x),&x\in {\mathbb {R}}, \end{aligned}$$

assume that the potential *V* in (5.1a) is one of the following:

(I): $$V(t,x)=0$$,
(II): $V(t,x)=\lambda (t)x$ with $\lambda :[0,\infty )\rightarrow {\mathbb {R}}$ continuous,
(III): $V(t,x)=\lambda (t)x^2$ with $\lambda :[0,\infty )\rightarrow {\mathbb {R}}$ continuous,
(IV): $V(t,x)=-\frac{l(l+1)}{\cosh ^2(x)}$ for some $l\in {\mathbb {N}}$.

Moreover, with ${\mathcal {O}},{\mathcal {U}}\subseteq {\mathbb {C}}$ such that ${\mathcal {U}}\subsetneqq {\mathcal {O}}$, and $\Omega \subseteq {\mathbb {C}}$ some open set containing the double sector $S_\alpha$ from (2.13) for some $\alpha \in (0,\frac{\pi }{2})$, we consider a family of analytic functions $\varphi _\kappa :\Omega \rightarrow {\mathbb {C}}$, $\kappa \in {\mathcal {O}}$, and initial conditions of the form

5.2 $$\begin{aligned} F_n(z)=\sum \limits _{l=0}^nC_l(n)\varphi _{\kappa _l(n)}(z),\qquad z\in \Omega , \end{aligned}$$

where $C_l(n)\in {\mathbb {C}}$, $\kappa _l(n)\in {\mathcal {U}}$. If we assume that

5.3 $$\begin{aligned} \lim \limits _{n\rightarrow \infty }\sup \limits _{z\in \Omega }|F_n(z)-\varphi _\kappa (z)|e^{-C|z|}=0, \end{aligned}$$

for some $C\ge 0$ and $\varphi _\kappa$ with $\kappa \in {\mathcal {O}}{\setminus }{\mathcal {U}}$, then there exists $T\in (0,\infty ]$ such that the sequence of solutions of (5.1) converge as

5.4 $$\begin{aligned} \lim \limits _{n\rightarrow \infty }\Psi (t,x;F_n)=\lim \limits _{n\rightarrow \infty }\sum \limits _{l=0}^nC_l(n)\Psi (t,x;\varphi _{\kappa _l(n)})=\Psi (t,x;\varphi _\kappa ) \end{aligned}$$

uniformly on compact subsets of $[0,T)\times {\mathbb {R}}$.

We point out that Theorem 5.1 shows (similarly as Theorem 4.5 and Remark 4.6) that for the potentials (I)–(IV), the supershift property of the initial datum $(F_n)_n$, with the stronger convergence (5.3), carries over to a supershift property of the solutions $(\Psi (t,x;F_n))_n$. Indeed, the convergence (5.3) implies the uniform convergence on all compact subsets of $\Omega$. Hence, $(F_n)_n$ admits a supershift according to Definition 4.1 in the metric space $X=\Omega$ with respect to the functions $\varphi _\kappa$. Furthermore, the convergence (5.4) shows the supershift property of the wave functions $(\Psi (t,x;F_n))_n$ in the metric space $X=[0,T)\times {\mathbb {R}}$ with respect to the functions $\phi _\kappa (t,x):=\Psi (t,x;\varphi _\kappa )$.

For the proof of Theorem 5.1, the Green’s functions for the respective potentials (I)–(IV) are investigated in the following paragraphs. The first potential is the free particle, where it is almost obvious that Assumption 3.1 is satisfied.

### (I) Free particle $V(t,x)=0$

We show that in this case the Green’s function

5.5 $$\begin{aligned} G(t,x,y)=\frac{1}{2\sqrt{i\pi t}}e^{-\frac{(y-x)^2}{4it}},\qquad t\in (0,\infty ),\,x,y\in {\mathbb {R}}, \end{aligned}$$

satisfies Assumption 3.1 with $T=\infty$. First of all it is clear that for every $t\in (0,\infty )$ and $x\in {\mathbb {R}}$, we can extend $G(t,x,\,\cdot \,)$ to an entire function by simply replacing $y\rightarrow z$. A direct computation shows that *G* satisfies the differential equation (3.6). Moreover, the Green’s function admits the decomposition (3.7) with

$$\begin{aligned} a(t)=\frac{1}{4t}\quad \text {and}\quad {\widetilde{G}}(t,x,z)=\frac{1}{2\sqrt{i\pi t}}. \end{aligned}$$

The bound (3.8) and the extension properties (3.9) are satisfied with the choices

$$\begin{aligned} A_0(t,x)=\frac{1}{2\sqrt{\pi t}}\quad \text {and}\quad B_0(t,x)=0. \end{aligned}$$

It is also clear that the limit condition (3.10) holds and the estimates for the derivatives in (3.11) follow immediately from the explicit form of ${\widetilde{G}}$.

### (II) Time-dependent uniform electric field $V(t,x)=\lambda (t)x$

It will be assumed that $\lambda :[0,\infty )\rightarrow {\mathbb {R}}$ is continuous. This type of potential was already investigated with respect to the time persistence of superoscillations; cf. [12, Theorem 3.6]. In the present setting, the Green’s function is of the form

5.6 $$\begin{aligned} G(t,x,y)=\frac{1}{2\sqrt{i\pi t}}e^{i\beta (t)+ixt\alpha '(t)+iy\alpha (t)-\frac{(y-x)^2}{4it}},\qquad t>0,\,x,y\in {\mathbb {R}}, \end{aligned}$$

where the coefficients $\alpha ,\beta :[0,\infty )\rightarrow {\mathbb {R}}$ are the solutions of the ordinary differential equations

5.7 $$\begin{aligned} t\alpha ''(t)+2\alpha '(t)=-\lambda (t)\quad \text {and}\quad \beta '(t)=-t^2\alpha '(t)^2,\qquad t>0, \end{aligned}$$

with initial conditions $\alpha (0)=\beta (0)=\lim _{t\rightarrow 0^+}t\alpha '(t)=0$. It will be shown that *G* satisfies Assumption 3.1 with $T=\infty$. As above, it is clear that for $t\in (0,\infty )$ and $x\in {\mathbb {R}}$, one can extend $G(t,x,\,\cdot \,)$ to an entire function by simply replacing $y\rightarrow z$. Differentiation and the use of the differential equations (5.7) show that *G* is indeed a solution of (3.6). Moreover, the Green’s function admits the decomposition (3.7) with

$$\begin{aligned} a(t)=\frac{1}{4t}\quad \text {and}\quad {\widetilde{G}}(t,x,z)=\frac{1}{2\sqrt{i\pi t}}e^{i\beta (t)+ixt\alpha '(t)+iz\alpha (t)}. \end{aligned}$$

It follows that the bound (3.8) and the extension properties (3.9) are satisfied with the choices

$$\begin{aligned} A_0(t,x)=\frac{1}{2\sqrt{\pi t}}\quad \text {and}\quad B_0(t,x)=|\alpha (t)|. \end{aligned}$$

Using the initial conditions of the coefficients $\alpha$ and $\beta$, one easily verifies that the limit condition (3.10) holds. Finally, when computing the derivatives $\frac{\partial }{\partial x}{\widetilde{G}}$, $\frac{\partial ^2}{\partial x^2}{\widetilde{G}}$, $\frac{\partial }{\partial t}{\widetilde{G}}$, one obtains functions of the form

$$\begin{aligned} P(t,x,z)e^{i\beta (t)+ixt\alpha '(t)+iz\alpha (t)}, \end{aligned}$$

where *P*(*t*, *x*, *z*) is continuous in *t* and *x*, and a polynomial in the *z*-variable. In this form it is not difficult to see that the derivatives can be estimated as in (3.11).

### (III) Time-dependent harmonic oscillator $V(t,x)=\lambda (t)x^2$

It will be assumed that $\lambda :[0,\infty )\rightarrow {\mathbb {R}}$ is continuous. In contrast to the potentials (I) and (II), it turns out that the expression for the Green’s function for the harmonic oscillator may only be valid on a finite time interval (0, *T*). It is of the form

5.8 $$\begin{aligned} G(t,x,y)=\frac{1}{2\sqrt{i\pi \alpha (t)}}e^{-\frac{\alpha '(t)x^2-2xy+\beta (t)y^2}{4i\alpha (t)}},\qquad t\in (0,T),\,x,y\in {\mathbb {R}}, \end{aligned}$$

where the coefficients $\alpha ,\beta$ are the solutions of the ordinary differential equations

5.9 $$\begin{aligned} \alpha ''(t)=-4\lambda (t)\alpha (t)\qquad \text {and}\qquad \beta ''(t)=-4\lambda (t)\beta (t), \end{aligned}$$

with initial conditions $\alpha (0)=\beta '(0)=0$ and $\beta (0)=\alpha '(0)=1$. It will be shown that *G* satisfies Assumption 3.1 with $T>0$ chosen as the smallest positive zero of either $\alpha$ or $\beta$, $T=\infty$ if $\alpha$ and $\beta$ have no positive zeros. With this choice of *T* it follows from the initial conditions that $\alpha (t)>0$ and $\beta (t)>0$ for $t\in (0,T)$. Again it is clear that for every $t\in (0,T)$ and $x\in {\mathbb {R}}$, one can extend $G(t,x,\,\cdot \,)$ to an entire function by simply replacing $y\rightarrow z$. Note that $\alpha$ and $\beta$ are linearly independent solutions of the differential equation (5.9) and hence the Wronskian has the constant value

$$\begin{aligned} \alpha '(t)\beta (t)-\alpha (t)\beta '(t)=1,\qquad t>0. \end{aligned}$$

Using this and (5.9) one verifies by a straightforward computation that *G* is a solution of (3.6). Moreover, the Green’s function admits the decomposition (3.7) with

$$\begin{aligned} a(t)=\frac{\beta (t)}{4\alpha (t)}\quad \text {and}\quad {\widetilde{G}}(t,x,z)=\frac{1}{2\sqrt{i\pi \alpha (t)}}e^{\frac{(\beta (t)-\alpha '(t))x^2+2xz(1-\beta (t))}{4i\alpha (t)}}. \end{aligned}$$

The bound (3.8) is satisfied with the choices

$$\begin{aligned} A_0(t,x)=\frac{1}{2\sqrt{\pi \alpha (t)}}\quad \text {and}\quad B_0(t,x)=\frac{|x||1-\beta (t)|}{2\alpha (t)}. \end{aligned}$$

Using $$\beta (0)=1$$ and

5.10 $$\begin{aligned} \lim \limits _{t\rightarrow 0^+}\frac{1-\beta (t)}{\alpha (t)}=\lim \limits _{t\rightarrow 0^+}\frac{-\beta '(t)}{\alpha '(t)}=\frac{0}{1}=0, \end{aligned}$$

it follows that the extension properties (3.9) hold. Using $\beta (0)=1$, the limit (5.10), as well as

$$\begin{aligned} \lim \limits _{t\rightarrow 0^+}\frac{\beta (t)-\alpha '(t)}{\alpha (t)}=\lim \limits _{t\rightarrow 0^+}\frac{\beta '(t)-\alpha ''(t)}{\alpha '(t)}=\lim \limits _{t\rightarrow 0^+}\frac{\beta '(t)+4\lambda (t)\alpha (t)}{\alpha '(t)}=0, \end{aligned}$$

one verifies that the limit condition (3.10) is satisfied. Finally, when computing the derivatives $\frac{\partial }{\partial x}{\widetilde{G}}$, $\frac{\partial ^2}{\partial x^2}{\widetilde{G}}$, $\frac{\partial }{\partial t}{\widetilde{G}}$, one obtains functions of the form

$$\begin{aligned} P(t,x,z)e^{\frac{(\beta (t)-\alpha '(t))x^2+2xz(1-\beta (t))}{4i\alpha (t)}}, \end{aligned}$$

where *P*(*t*, *x*, *z*) is continuous in *t* and *x*, and a polynomial in the *z*-variable. In this form, it is not difficult to see that the derivatives can be estimated as in (3.11).

#### Remark 5.2

For the special case of a time-independent harmonic oscillator, this potential was already investigated with respect to the evolution of superoscillations in [36, Proposition 5.2]. The above considerations can be viewed as a time-dependent generalization of the earlier results. Note that in the particular situation $V(t,x)=\omega ^2x^2$ with $\omega >0$, the Green’s function (5.8) reduces to

$$\begin{aligned} G(t,x,y)=\frac{\sqrt{\omega }}{\sqrt{2i\pi \sin (2\omega t)}}e^{-\frac{\omega (y-x)^2}{2i\tan (2\omega t)}-i\omega xy\tan (\omega t)},\qquad t\in \Big (0,\frac{\pi }{4\omega }\Big ),\,x,y\in {\mathbb {R}}, \end{aligned}$$

and for $V(t,x)=-\omega ^2x^2$ with $\omega >0$, the Green’s function (5.8) becomes

$$\begin{aligned} G(t,x,y)=\frac{\sqrt{\omega }}{\sqrt{2i\pi \sinh (2\omega t)}}e^{-\frac{\omega (y-x)^2}{2i\tanh (2\omega t)}+i\omega xy\tanh (\omega t)},\qquad t\in (0,\infty ),\,x,y\in {\mathbb {R}}. \end{aligned}$$

### (IV) Pöschl–Teller potential $V(t,x)=-\frac{l(l+1)}{\cosh ^2(x)}$, $l\in {\mathbb {N}}$

For the Pöschl–Teller potential, it turns out that the Green’s function cannot be extended to the whole complex plane, due to singularities on the imaginary axis. For $x,y\in {\mathbb {R}}$ and $t>0$, the Green’s function is given by

5.11 $$\begin{aligned} G(t,x,y)=\bigg (\frac{1}{2\sqrt{i\pi t}}+\sum \limits _{m=1}^l\frac{m(l-m)!}{2(l+m)!}Q_l^m(x)Q_l^m(y)R\big (m^2t,m(y-x)\big )\bigg )e^{-\frac{(y-x)^2}{4it}},\nonumber \\ \end{aligned}$$

where we use the function

5.12 $$\begin{aligned} R(t,z):= e^z\Lambda \Big (\frac{z}{2\sqrt{it}}-\sqrt{it}\Big )-e^{-z}\Lambda \Big (\frac{z}{2\sqrt{it}}+\sqrt{it}\Big ),\qquad t>0,\,z\in {\mathbb {C}},\nonumber \\ \end{aligned}$$

with $\Lambda (z):= e^{z^2}(1-{\text {erf}}(z))$ a modification of the error function,

$$Q_l^m(x):= P_l^m(\tanh (x))$$

and $P_l^m$ the associated Legendre polynomials. This Green’s function can for example be found in [39, Section 6.6.3]. It follows from the Legendre differential equation [1, Eq. 8.1.1] that $Q_l^m$ satisfies

5.13 $$\begin{aligned} (Q_l^m)''(x)+\Big (\frac{l(l+1)}{\cosh ^2(x)}-m^2\Big )Q_l^m(x)=0,\qquad x\in {\mathbb {R}}. \end{aligned}$$

Due to the representations [1, Equations 8.6.6 & 8.6.18], we know that the associated Legendre polynomials are of the form

$$\begin{aligned} P_l^m(\xi )=(1-\xi ^2)^{\frac{m}{2}}\Big (\text {polynomial in }\xi \Big ),\qquad \xi \in (-1,1). \end{aligned}$$

From this, it follows that also $Q_l^m$ is of the form

5.14 $$\begin{aligned} Q_l^m(x)=\frac{1}{\cosh ^m(x)}\Big (\text {polynomial in }\tanh (x)\Big ),\qquad x\in {\mathbb {R}}. \end{aligned}$$

In particular, it is possible to extend $Q_l^m$ and hence also $G(t,x,\,\cdot \,)$ analytically to the complex domain ${\mathbb {C}}{\setminus } i\pi ({\mathbb {Z}}+\frac{1}{2})$, where only the zeros of the function $\cosh$ were excluded, that is, we consider *G*(*t*, *x*, *z*) in (5.11) with *y* replaced by $z\in {\mathbb {C}}{\setminus } i\pi ({\mathbb {Z}}+\frac{1}{2})$. Moreover, it can easily be checked that

5.15 $$\begin{aligned} \begin{aligned} \frac{\partial }{\partial z}R(t,z)&=\frac{z}{2it}R(t,z)-\frac{2}{\sqrt{i\pi t}}\sinh (z), \\ \frac{\partial }{\partial t}R(t,z)&=i\Big (1+\frac{z^2}{4t^2}\Big )R(t,z)+\frac{z\sinh (z)}{t\sqrt{i\pi t}}+\frac{2i\cosh (z)}{\sqrt{i\pi t}}. \end{aligned} \end{aligned}$$

If one uses the derivatives (5.13) and (5.15) together with the identity

$$\begin{aligned} \sum \limits _{m=1}^l\frac{m(l-m)!}{(l+m)!}Q_l^m(z)\sinh \big (m(z-x)\big )Q_l^m(x)=\frac{l(l+1)}{4}\big (\tanh (z)-\tanh (x)\big ) \end{aligned}$$

for the Legendre polynomials, a straightforward (but long and technical) computation shows that (5.11) satisfies the Schrödinger equation (3.6).

The Green’s function admits the decomposition (3.7) with $a(t)=\frac{1}{4t}$ and

$$\begin{aligned} {\widetilde{G}}(t,x,z)=\frac{1}{2\sqrt{i\pi t}}+\sum \limits _{m=1}^l\frac{m(l-m)!}{2(l+m)!}Q_l^m(x)Q_l^m(z)R\big (m^2t,m(z-x)\big ). \end{aligned}$$

Now let us assume that the domain $\Omega$ of Theorem 5.1 has a positive distance to the poles $i\pi ({\mathbb {Z}}+\frac{1}{2})$. If not, we can always shrink it, such that it has positive distance to $i\pi ({\mathbb {Z}}+\frac{1}{2})$ and still contains the double sector $S_\alpha$, e.g. by taking the intersection with

$$\begin{aligned} \Omega _\alpha :=\left\{ z\in {\mathbb {C}} \,\Big |\, \vert {\text{ Im }}(z)\vert <\tan (\alpha )|{\text{ Re }}(z)|+\frac{\pi }{4}\right\} . \end{aligned}$$

Since the domain $\Omega$ now has positive distance to the zeros of $\cosh (z)$, we can use

$$\begin{aligned} |\cosh (z)|^2=\sinh ^2({\text {Re}}z)+\cos ^2({\text {Im}}z)\ge c>0,\qquad z\in \Omega , \end{aligned}$$

to estimate

5.16 $$\begin{aligned} |Q_l^m(z)|\le A_l^me^{B_l^m|z|},\qquad z\in \Omega , \end{aligned}$$

for some $A_l^m,B_l^m\ge 0$. Due to the properties [4, Lemma 2.1] of the function $\Lambda$, we can also estimate (5.12) as

5.17 $$\begin{aligned} |R(t,z)|\le 2\Lambda \Big (-\frac{\sqrt{t}}{\sqrt{2}}\Big )e^{|{\text {Re}}(z)|},\qquad t>0,\,z\in {\mathbb {C}}; \end{aligned}$$

to verify this inequality, it suffices to consider ${\text {Re}}(z)+{\text {Im}}(z)\ge 0$ due to the symmetry $R(t,-z)=R(t,z)$ and to use the estimate as well as the monotonicity of $\Lambda$. Summing up, we conclude that for $t>0$, $x\in {\mathbb {R}}$ and $z\in \Omega$, the function ${\widetilde{G}}$ can be estimated as

$$\begin{aligned} |{\widetilde{G}}(t,x,z)|\le \frac{1}{2\sqrt{\pi t}}+\sum \limits _{m=1}^l\frac{m(l-m)!}{(l+m)!}(A_l^m)^2\Lambda \Big (-\frac{m\sqrt{t}}{\sqrt{2}}\Big )e^{(m+B_l^m)(|x|+|z|)}, \end{aligned}$$

from which the bound (3.8) follows if we choose the coefficients

$$\begin{aligned} A_0(t,x)&=\frac{1}{2\sqrt{\pi t}}+\sum \limits _{m=1}^l\frac{m(l-m)!}{(l+m)!}(A_l^m)^2e^{(m+B_l^m)|x|}\Lambda \Big (-\frac{m\sqrt{t}}{\sqrt{2}}\Big ), \\ B_0(t,x)&=\max \limits _{1\le m\le l}(m+B_l^m). \end{aligned}$$

Using $\Lambda (0)=1$, it is not difficult to see that these coefficients can be continuously extended as in (3.9). For the limit (3.10), we use the asymptotics

$$\begin{aligned} R(t,z)=\frac{4\sinh (z)\sqrt{it}}{z\sqrt{\pi }}+{\mathcal {O}}(t),\quad \text {as }t\rightarrow 0^+,\quad \forall z\in {\mathbb {C}}, \end{aligned}$$

which follows from [4, Lemma 2.1]. Finally, using the derivative of the function $\Lambda$ from [4, Lemma 2.1] and the fact that $Q_l^m$ contains only powers of the functions $\cosh$ and $\sinh$, as well as the inequalities (5.16) and (5.17), one can show that also the derivatives of ${\widetilde{G}}$ are exponentially bounded as required in (3.11).
